# Molecular Mechanism of Rheumatic Diseases and Efficacy of Current Therapies

**DOI:** 10.1155/2017/2401329

**Published:** 2017-03-15

**Authors:** Sadiq Umar, Abdul M. Tyagi, Anil K. Singh, Abdul Haseeb

**Affiliations:** ^1^Lillehei Heart Institute, Division of Cardiology, University of Minnesota, Minneapolis, MN 55455, USA; ^2^Division of Endocrinology, Metabolism and Lipid, School of Medicine, Emory University, Atlanta, GA, USA; ^3^College of Pharmacy, Washington State University, Spokane, WA, USA; ^4^Department of Cellular and Molecular Medicine, Lerner Research Institute, Cleveland Clinic, Cleveland, OH 44195, USA

Arthritis represents one of the most prevalent chronic health problems and is a leading cause of disability; it was 52.5 million Americans in 2010–12 with an estimate of 78 million by 2040 where two-thirds will be women, suffering from this disease. Arthritis includes more than 100 rheumatic diseases' condition which affects joint and tissue which surround the joint and other connective tissue, the most common being osteoarthritis which affects around 30 million US adults while others include juvenile arthritis, fibromyalgia, gout, rheumatoid arthritis, and systemic lupus erythematosus (SLE). It occurs often in people with chronic conditions, such as heart disease and diabetes, as well as those who are obese.

Antigen-activated CD4^+^ T cells stimulate monocytes, macrophages, and synovial fibroblasts to produce the cytokines interleukin-1*β* (IL-1*β*), IL-6, and tumor necrosis factor-*α* (TNF-*α*). These cytokines act as potent inducer of inflammatory responses through upregulation of many genes, including cytokines, chemokines, and adhesion molecules. The focus of treatment for arthritis is to control pain, minimize joint damage, and improve or maintain function and quality of life. Current treatment modalities for rheumatic diseases either produce symptomatic relief (NSAIDs) or modify the disease process (DMARDs) and biological agents, mainly TNF blockers. Other potential experimental promising therapies are IL-17 blockers and IL-23 blockers. Though effective, their use is also limited by cost and their side effects. As a result, the interest in alternative, well tolerated anti-inflammatory remedies has reemerged. Targeting the pathogenic pathway of chronic inflammation represents an unmet challenge for controlling disease activity, preventing functional disability and maintaining an adequate quality of life in patients with rheumatic diseases.

In this special issue, we invited the researchers to contribute their work in understanding rheumatic disease to better take care of the people affected by these disease. P. Klinger et al. investigate the role of pigment epithelium-derived factor PEDF on the genome-wide gene expression, a pluripotent protein expressed in multiple tissues and involved in multiple signaling pathways, including the IP3-AKT, MEK-ERK, or PLA2-PPAR pathway, and showed it as marker and future therapy to stabilize the chondrocyte phenotype of articular cartilage and to prevent its degradation. I. P. Perpétuo et al. studied the role of TNF inhibitors (TNFi) in the differentiation and activity of OC in rheumatoid arthritis (RA) patients. They proposed that TNFi arrests bone loss and erosions, either by direct reduction of osteoclast precursor numbers or by inhibiting of intracellular signaling pathways acting through TRAF6. C. Lin et al. present their findings in the paper “Gray Matter Atrophy within the Default Mode Network of Fibromyalgia: A Meta-Analysis of Voxel-Based Morphometry Studies.” E. V. Zakharova et al. published their finding in the paper “Immunosuppressive Treatment for Lupus Nephritis: Long-Term Results” in 178 patients. A. J. Ruiz-Padilla et al. published “The -174G/C Interleukin-6 Gene Promoter Polymorphism as a Genetic Marker of Differences in Therapeutic Response to Methotrexate and Leflunomide in Rheumatoid Arthritis.”

Y.-F. Liu et al. showed that the effect of berberine alleviates monosodium urate crystals-induced inflammation by downregulating NLRP3 and IL-1*β* expressions and connects it with inactivation of NLRP3 inflammasome. W. Hu et al. showed their analysis “Association between Toll-Like Receptor 4 Polymorphisms and Systemic Lupus Erythematosus Susceptibility: A Meta-Analysis.” S. Kotake et al. published “Elevated Ratio of Th17 Cell-Derived Th1 Cells (CD161^+^Th1 Cells) to CD161^+^Th17 Cells in Peripheral Blood of Early-Onset Rheumatoid Arthritis Patients.”

We hope this special issue covered many important aspects in current updates and therapeutics strategies for rheumatic diseases, which will surely provide us with a better understanding about the pathogenesis, diagnosis, and treatment of these rheumatic diseases.

*Sadiq Umar* *Abdul M. Tyagi* *Anil K. Singh* *Abdul Haseeb*

